# Heatwaves and mortality in Queensland 2010–2019: implications for a homogenous state-wide approach

**DOI:** 10.1007/s00484-023-02430-6

**Published:** 2023-02-03

**Authors:** Richard C. Franklin, Hannah M. Mason, Jemma C. King, Amy E. Peden, John Nairn, Lauren Miller, Kerrianne Watt, Gerard FitzGerald

**Affiliations:** 1 Discipline of Public Health and Tropical Medicine, CPHMVS, James Cook University, Townsville, QLD 4811 Australia; 2 School of Population Health, Faculty of Medicine and Health, University of New South Wales, Sydney, NSW Australia; 3 School of Biological Sciences, Faculty of Sciences, Engineering and Technology, The University of Adelaide, Adelaide, SA Australia; 4 Information Support, Research & Evaluation, Queensland Ambulance Service, Brisbane, QLD Australia; 5 School of Public Health and Social Work, Faculty of Health, Queensland University of Technology, Sydney, NSW Australia

**Keywords:** Heatwave, Climate change, Deaths, Australia, Excess Heat Factor, Public health

## Abstract

Heatwaves are a significant cause of adverse health outcomes and mortality in Australia, worsening with climate change. In Queensland, the northeastern-most state, little is known about the impact of heatwaves outside of the capital city of Brisbane. This study aims to explore the impact of heatwaves on mortality across various demographic and environmental conditions within Queensland from 2010 to 2019. The Excess Heat Factor was used to indicate heatwave periods at the Statistical Area 2 (SA2) level. Registered deaths data from the Australian Bureau of Statistics and heatwave data from the Bureau of Meteorology were matched using a case-crossover approach. Relative risk and 95% confidence intervals were calculated across years, regions, age, sex, rurality, socioeconomic status, and cause of death. Heatwaves were associated with a 5% increase in all-cause mortality compared to deaths on non-heatwave days, with variability across the state. The risk of death on a heatwave day versus a non-heatwave day varied by heatwave severity. Individuals living in urban centers, the elderly, and those living in regions of lower socioeconomic status were most impacted by heatwave mortality. The relative risk of dying from neoplasms, nervous system conditions, respiratory conditions, and mental and behavioral conditions increased during heatwaves. As heatwaves increase in Queensland due to climate change, understanding the impact of heatwaves on mortality across Queensland is important to tailor public health messages. There is considerable variability across communities, demographic groups, and medical conditions, and as such messages need to be tailored to risk.

**Supplementary Information:**

The online version contains supplementary material available at 10.1007/s00484-023-02430-6.

## Introduction

It is well known that exposure to extreme weather conditions is increasing globally and impacting the health and wellbeing of those exposed (Akhtar 2020; Campbell et al. 2018; Chesnais et al. 2019). Of all types of extreme weather, heatwaves contribute to more deaths in Australia than any other environmental disaster (Scalley et al. 2015; Chesnais et al. 2019; Coates et al. 2014). Heat-health impacts are considered largely preventable (Nitschke et al. 2016), and the examination of previous heat events can provide relevant evidence for policy and strategy development as well as public health communication (Williams et al. 2011). Several governments are working on or have developed mitigation and adaptation strategies to address the impacts of heatwaves on health systems (Bureau of Meteorology; Scalley et al. 2015). One of the challenges for governments, especially those who have large landmass areas, is the non-homogenous nature of heatwaves and variability in peak temperatures from coast to inland, complicating government and health system planning (Trancoso et al. 2020).

**Fig. 1:**
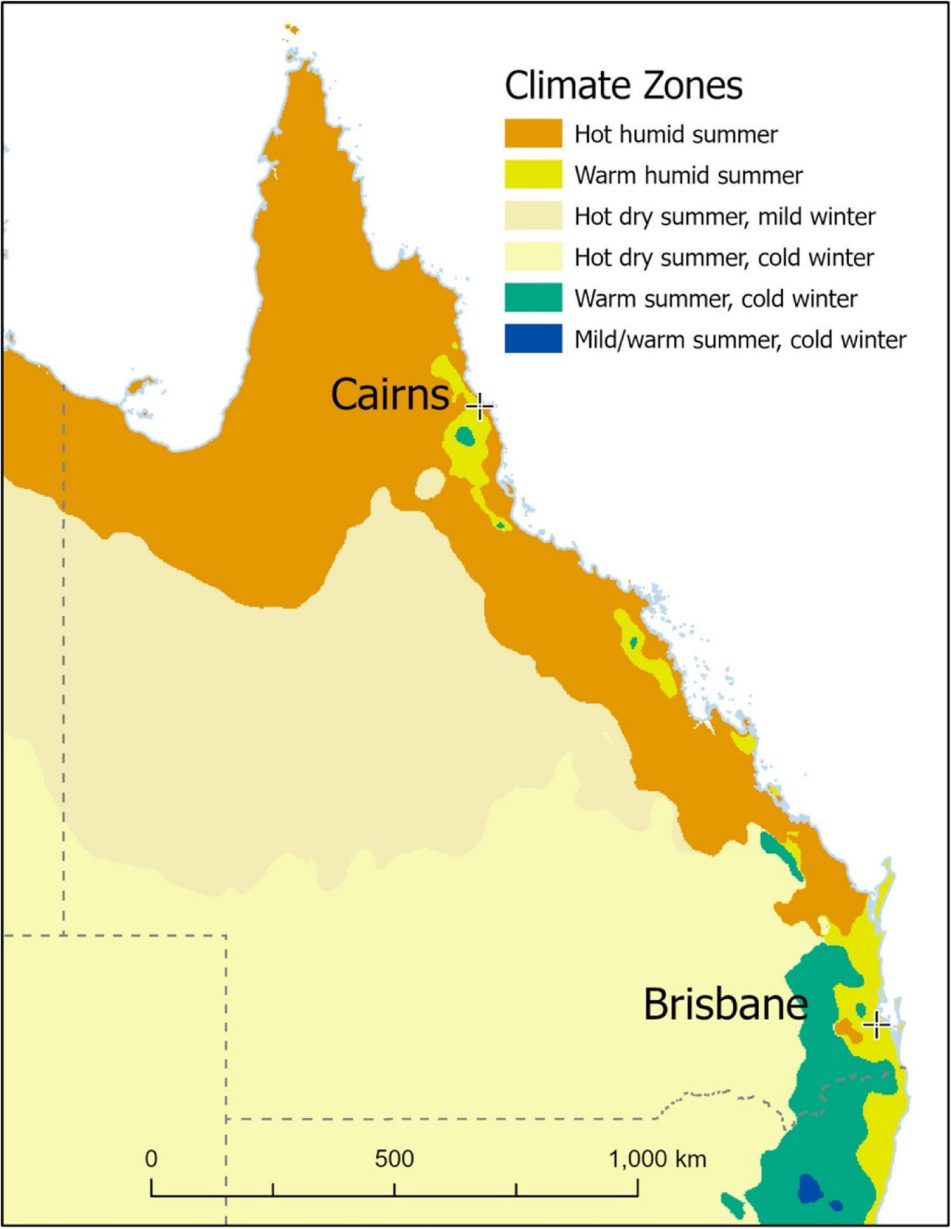
Queensland climate zones based on temperature and humidity (Bureau of Meteorology n.d.)

Queensland is the northeastern most state of Australia and has multiple climatic regions (Fig. 1), stretching from hot and humid in the tropical north, sub-tropical temperatures in the south, hot arid in the western interior, and cool and temperate in the southeastern belt just inland from the coast (Business Queensland 2021a). It is estimated that peak heatwave temperatures will rise 3.2 °C from 1986–2005 to 2081–2099 across the state, with sharp intensification of heatwaves from 2040 onwards (Trancoso et al. 2020). Human-induced climate change is projected to increase the duration, frequency, severity, and spread of heatwaves in the absence of strong mitigation measures (Chesnais et al. 2019; Masson-Delmotte et al. 2021). The true impact of heatwaves on deaths in Queensland is not clearly described in the literature, and that which does has a primary focus on the capital city of Brisbane (Mason et al. 2022). Queensland is a decentralized state with a disaggregated population spread compared to other states and territories (Dezuanni et al. 2018). While there is a greater population density in and around the capital, little is known about the impacts of heatwaves outside of this area, with policy needing to move away from a one-size-fits-all approach to be effective across the whole of the state. Within Queensland, there are 77 local government areas, ranging in size geographically and population (Business Queensland 2021b). This provides an opportunity to examine the impact of heatwaves across diverse demographic and meteorological environments to understand further the unique geographic, demographic, and population health challenges that heatwaves represent in each of these regions.

### Impact of heatwaves on deaths

The relationship between mortality and temperature exhibits a U-shaped curve that has been observed globally, with more deaths occurring in periods of sustained, abnormally hot or cold temperatures (Jegasothy et al. 2017). Prolonged exposure to extreme heat can lead to direct heat-related mortality such as thermoplegia, heatstroke, heat cramps, and heat syncope, and can also cause indirect effects primarily through dehydration, including cardiovascular, respiratory, and renal conditions (Guo et al. 2018; Mason et al. 2022). Previous research has indicated that the risk of mortality in Brisbane (a metropolitan region and the capital city of Queensland, Australia) ranges from 5 to 72% higher during heatwave periods versus non-heatwave periods (Wang et al. 2015; Tong et al. 2014b, 2012). Additionally, older adults have been found to be at higher risk of heatwave morality in Brisbane in comparison to their younger counterparts (Tong et al. 2014a; Wang et al. 2012, 2015). It is also noted in the literature that those with pre-existing conditions including cardiovascular diseases (Wang et al. 2012, 2015), diabetes (Wang et al. 2012), and Alzheimer’s (Xu et al. 2019) were at a higher risk of dying during Brisbane heatwaves. The impact of heatwaves on mortality across the whole of Queensland remains unknown, a gap of this research will address.

### Defining heatwaves

**Fig. 2:**
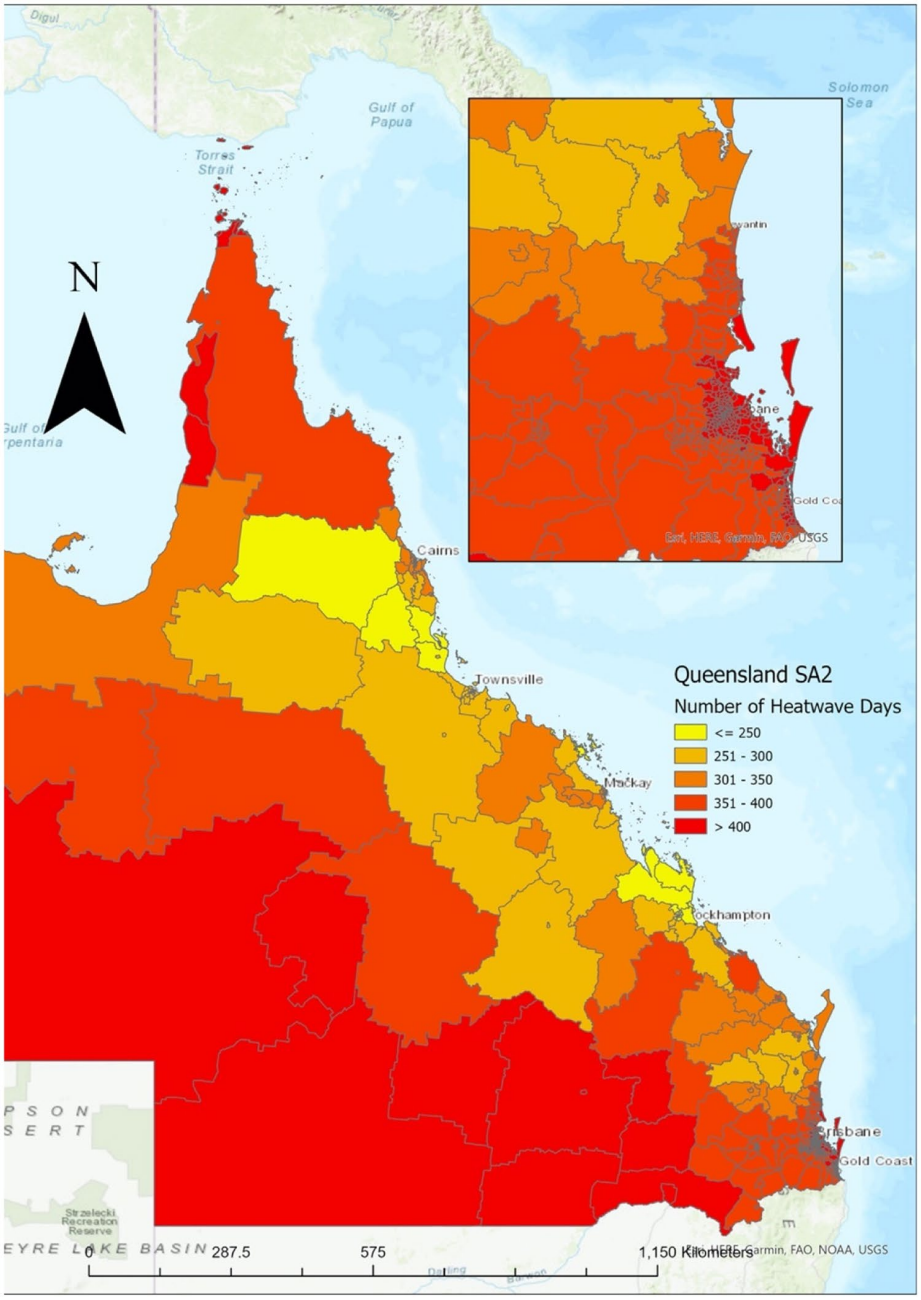
Number of heatwave days per Statistical Area Level 2, Queensland 2010–2019

**Fig. 3:**
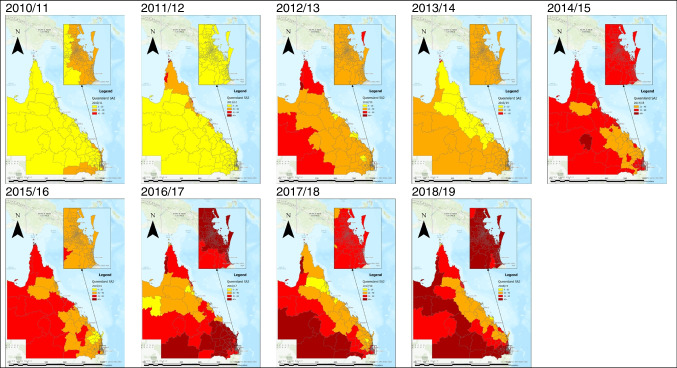
Heatwave maps per Statistical Area Level 2 across Queensland from 2010/11 to 2018/19 financial years. Note: Australian financial year is from 1 July – 30th June. Inset map pictures South-East Queensland. Full size images available in Supplementary File 1

The extent of the relationship between heatwaves and mortality is varied by heatwave definition (Tong et al. 2014b). It also varies according to the prevailing environmental conditions and the demographic and socioeconomic circumstances that enable adaptation to those prevailing conditions. Currently, there is no standardized definition for heatwaves in Australia across jurisdictions (Mason et al. 2022; Scalley et al. 2015), although it is noted that there has been a move by the Bureau of Meteorology to use the Excess Heat Factor (EHF) (Nairn and Fawcett 2014, 2017). The EHF, a metric developed by Nairn and Fawcett, is used to identify and measure the severity of heatwaves (Nairn and Fawcett 2015, 2017). This metric was recently used in the *Reducing Illness and Lives Lost from Heatwaves* Report, which explored heatwave morbidity and mortality across Australia (Physical Environment Research Network 2021). To improve cross-literature comparisons, this study will use the Excess Heat Factor to indicate periods of prolonged heat (Nairn and Fawcett 2015, 2017). The EHF takes the prevailing environmental conditions into account and thus measures “variation from normal” as a measure of environmental stress (for EHF formula please see https://doi.org/10.3390/ijerph120100227). EHF was designed to capture heatwave intensity as it applies to health outcomes (Nairn and Fawcett 2014) and is based on a 3-day daily averaged temperature in relation to the 95th percentile of long-term average daily temperatures, and the prior 30-day daily temperatures in a given location (Varghese et al. 2019). The total number of heatwave days across all regions in Queensland over the 10-year period according to the EHF is mapped in Fig. 2 and mapped over each financial year in Fig. 3.

The EHF defines three intensities of heatwaves: low intensity, severe, and extreme (Nairn and Fawcett 2014, 2017). During heatwaves of low severity (EHF values less than 1), it is expected that most will be able to cope, at severe intensities (EHF values greater than 1 and less than 3), vulnerable populations are challenged, and at extreme intensities (EHF values greater than 3), everyone is at risk and infrastructure may be impacted (including power and transport) (Chesnais et al. 2019; Nairn and Fawcett 2014, 2017). Given a 3-day average is used for the EHF, the severity of a heatwave at its most extreme point determines the severity of the heatwave on the 2 days preceding it (Nairn and Fawcett 2014, 2017).

### Aims and objectives

This study contributes to the Heat, Health, and Human Environment Project funded by the Queensland Department of Environment and Science. This research aims to improve understanding of how individual health and health service demand is affected by heatwaves at a state-wide level.

## Materials and methods

### Setting and participants

This study was conducted in the state of Queensland in Australia. Queensland is the third most populated state in Australia, with an estimated resident population of 5,206,400 in 2021 (Australian Bureau of Statistics 2021). Queensland represents 22.5% of Australia’s mainland area at 1.72 million km^2^ (Geoscience Australia, n.d.). The median age of Queenslanders is 37, with 19.4% under 14 years old and 15.3% over 65 years old (Australian Bureau of Statistics 2017). The health system is made up of a combination of public and private health services funded through a combination of the national compulsory health insurance scheme (Medicare), private health insurance, and individual co-payments. Health service availability differs across the state, with 38% of the population living away from tertiary hospital facilities (eHealth Queensland 2021).

### Data collection

The study compared all-cause mortality data with environmental data at the local level. Heatwave data were provided by the Bureau of Meteorology, and data items included: EHF values, SA2 region, and calendar date. All-cause mortality data for Queensland from 1st January 2010 to 31st December 2019 was supplied from the Australian Bureau of Statistics (ABS; ICD-10: A00-Z99). The dataset included information on usual area of residence, age, sex, Indigenous status, underlying cause of death, and other causes of death. Usual area of residence was provided at the Statistical Areas Level 2 level (SA2) which is defined by the ABS as a functional area with an average of approximately 10,000 people, with a minimum of 3000 and a maximum of 25,000 people and represents communities that interact together economically and socially (Australian Bureau of Statistics 2016b).

Rurality was derived from SA2 level information using the Australian Statistical Geography Standard (ASGS), a measure based on relative access to services (Australian Bureau of Statistics 2016a). SA2 regions were categorized as major cities, inner regional, outer regional, remote, or very remote (Australian Bureau of Statistics 2016a). Similarly, socio-economic status was determined on the SA2 level based on the Index of Socio-economic Advantage and Disadvantage (IRSAD), with low scores indicating greater disadvantage and high scores indicating greater advantage in general. IRSAD categories were grouped into low (deciles 1–3), medium (deciles 4–7), and high (deciles 8–10).

As the data were collected over a 10-year period, there were some changes in SA2 designation. EHF values were recombined and averaged for regions that were split at some time over the 10-year period. SA2 regions that had a change in rurality designation were coded as their most current designation according to the ASGS.

### Data analysis

A case-crossover approach was undertaken to analyze the relationship between heatwaves and deaths in Queensland. Heatwave dates and death dates were converted to “day of the year” (from 1 to 366) for data matching purposes. Number of deaths that occurred on days labeled as “heatwave” were compared with number of deaths that occurred on the same day of the calendar year on which a heatwave did not occur (matched), for all-cause mortality (all age groups), and for each age-group, and separately for broad disease groups as defined by International Classification of Disease version 10 chapters (World Health Organization 2015).

Regions were categorized into the rurality groupings using the Australian Statistical Geography Standard (ASGS) Remoteness Structure (Australian Bureau of Statistics 2011). The Index of Relative Socio-economic Advantage and Disadvantage (IRSAD) was used to classify regions socioeconomically, a measure that is determined by a range of factors such as an individual’s income, qualifications, and occupation (Australian Bureau of Statistics 2018). Those in the 8–10th decile were considered highly advantaged, those in the 4–7th decile were considered middle advantage, and those in the 1–3rd decile were considered to be from a low level of advantage (Australian Bureau of Statistics 2018). Due to low numbers, causes of death including eye, ear and mastoid, pregnancy and childbirth, and symptoms and other not elsewhere classified were grouped together as “other.”

Data were processed using IBM Statistical Package for Social Sciences SPSS Version 28. Relative risk (RR) and 95% confidence intervals were calculated using Microsoft Excel (*α* = 0.05). To analyze deaths across a 10-year period, years were converted into financial years (1 July–30 June) so that drop-off rates from registration lags common at the end of the calendar year did not affect analysis, and to capture warmer months of Australia within the season (summer is from December to February).

Relative risk was calculated by the following formula:

$$\mathrm{Relative}\;\mathrm{risk}=\frac{\mathrm{number}\;\mathrm{of}\;\mathrm{deaths}\;\mathrm{on}\;\mathrm{heatwave}\;\mathrm{days}\div\mathrm{number}\;\mathrm{of}\;\mathrm{heatwave}\;\mathrm{days}}{\mathrm{number}\;\mathrm{of}\;\mathrm{deaths}\;\mathrm{on}\;\mathrm{non}\;\mathrm{heatwave}\;\mathrm{days}\div\mathrm{number}\;\mathrm{of}\;\mathrm{non}\;\mathrm{heatwave}\;\mathrm{days}}$$

Maps and spatial data were generated using the ArcGIS Pro software. A Queensland shapefile was derived from the Australia-wide ASGS Edition 2016 shapefile obtained from the Australian Bureau of Statistics (https://abs.gov.au; 1270.0.55.001). The number of heatwave days per financial year, total number of heatwave days, and relative risk for deaths were tabulated on the SA2 level in Microsoft Excel and then imported into the ArcGIS software to generate the maps.

## Results

**Fig. 4:**
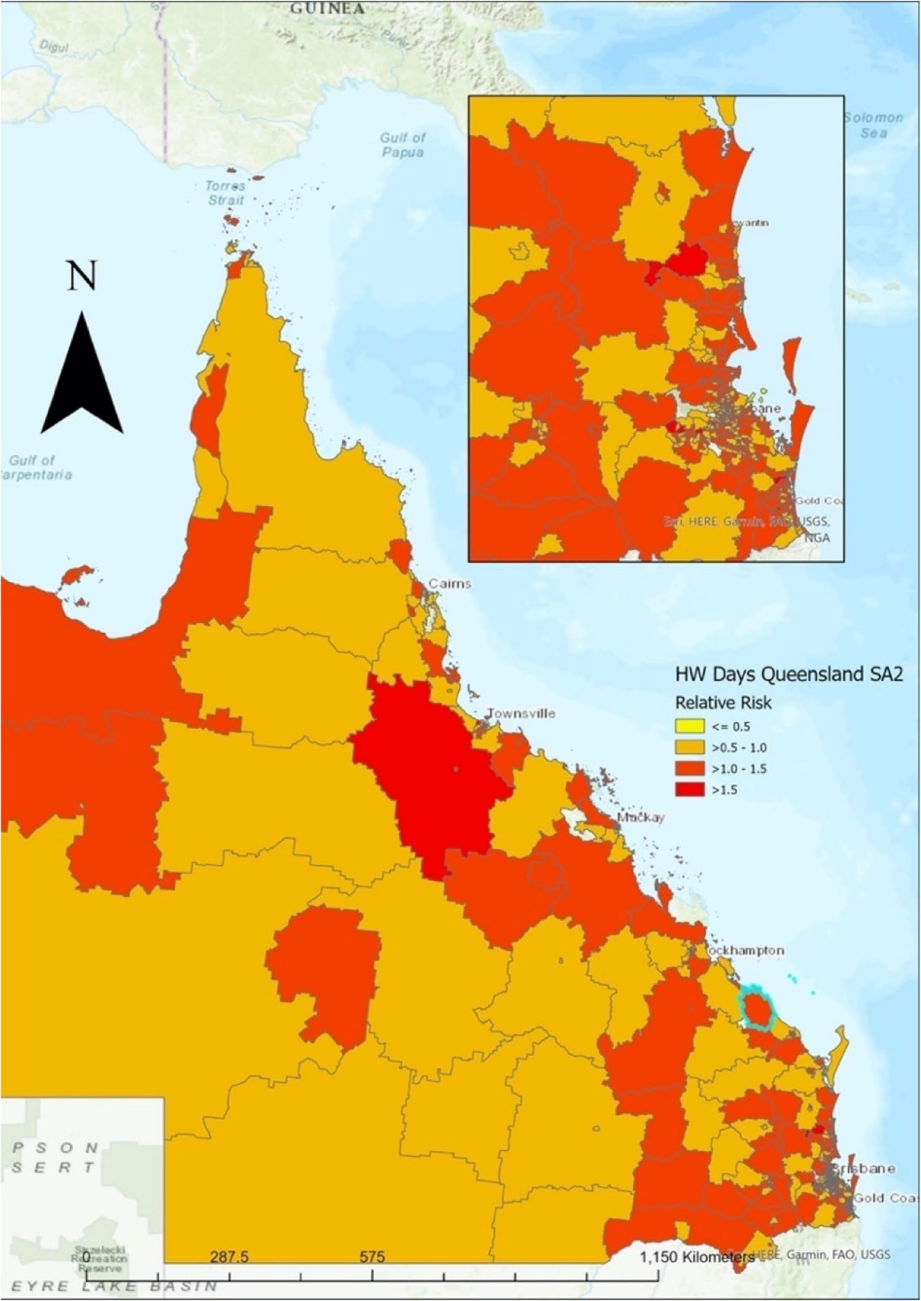
Relative risk of mortality during heatwaves across Queensland from 2010 to 2019

Over the study period, of the matched cases (those that occurred on a day where a heatwave occurred over the 10-year period), there were 99,118 deaths in Queensland across 528 SA2 regions (28,142 deaths occurred on 123,138 heatwave days and 70,976 deaths occurred on 325,722 non-heatwave days). Overall, the total risk of mortality was 5% greater during heatwaves from 1st July 2010–30th January 2019 (*RR* = 1.05, *CI*: 1.03–1.06). This effect was varied across regions (Fig. 4).

**Fig. 5:**
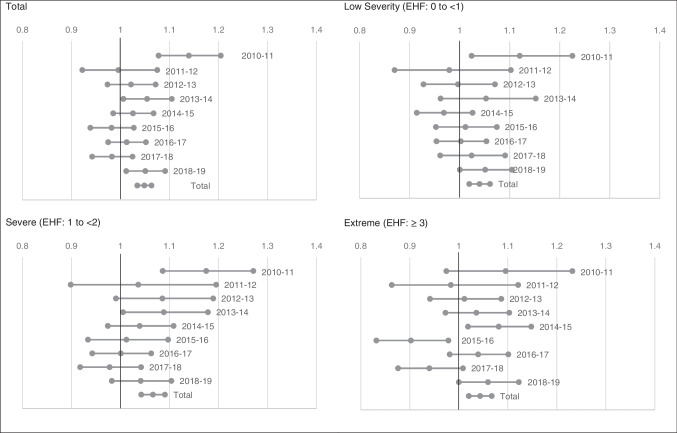
Relative risk for total, low, severe, and extreme heatwaves for matched days

Heatwaves significantly increased the risk of mortality in the 2010/11 (*RR* = 1.14, *CI*: 1.08–1.20), 2013/14 (*RR* = 1.05, *CI*: 1.01–1.10), and 2018/19 (*RR* = 1.05, *CI*: 1.01–1.09) financial years. The impact of heatwave severity on mortality varied across the financial years. Extreme heatwaves were significantly inversely associated with mortality in the 2015/16 (*RR* = 0.90, *CI*: 0.83–0.98) financial year (Fig. 5).

Heatwaves did not significantly increase the risk of mortality for Aboriginal and Torres Strait Islander Peoples (*RR* = 1.03, *CI*: 0.95–1.12), but increased the risk for non-Aboriginal people (*RR* = 1.05, *CI*: 1.04–1.07). For those in which Aboriginal and Torres Strait Islander status was not stated, heatwaves were inversely associated (*RR* = 0.74, *CI*: 0.64–0.87).

The risk of mortality was significantly higher during heatwaves for those 65–79 years old (*RR* = 1.06, *CI*: 1.03–1.08) and 80 + (*RR* = 1.06, *CI*: 1.04–1.08). The risk of mortality among older people increased in all categories of severity of heatwaves. For low severity heatwaves, the risk of mortality for those who were 60–64 years old (*RR* = 1.07, *CI*: 1.01–1.13), 65–79 years old (*RR* = 1.05, *CI*: 1.01–1.09), and 80 + (*RR* = 1.03, *CI*: 1.00, 1.06). Severe heatwaves increased the risk of mortality for those 65–79 years old (*RR* = 1.09, *CI*: 1.04–1.13) and 80 + (*RR* = 1.09, *CI*: 1.06–1.13) but were inversely associated for those 0–4 years old (*RR* = 0.75, *CI*: 0.59, 0.96). Extreme heatwaves significantly increased the risk of mortality for those over 80 years old (*RR* = 1.06, *CI*: 1.03–1.09) (Table 1). No statistically significant increased risk for children was found.

**Table 1:** Risk of mortality for demographic groups by heatwave severity (Queensland; 2010–2019)

			Relative risk (95% confidence interval)		
		Low	Severe	Extreme	Total
Age	0–4 years	0.82 (0.67, 1.01)	**0.75 (0.59, 0.96)**	1.06 (0.86, 1.30)	0.87 (0.76, 1.00)
	5–19 years	0.96 (0.73, 1.26)	1.11 (0.83, 1.49)	0.87 (0.63, 1.20)	0.98 (0.81, 1.18)
	20–59 years	1.05 (0.97, 1.14)	1.06 (0.97, 1.15)	1.01 (0.93, 1.10)	1.04 (0.99, 1.10)
	60–64 years	**1.07 (1.01, 1.13)**	0.96 (0.90, 1.02)	1.01 (0.95, 1.08)	1.02 (0.98, 1.06)
	65–79 years	**1.05 (1.01, 1.09)**	**1.09 (1.04, 1.13)**	1.04 (1.00, 1.08)	**1.06 (1.03, 1.08)**
	80 + years	**1.03 (1.00, 1.06)**	**1.09 (1.06, 1.13)**	**1.06 (1.03, 1.09)**	**1.06 (1.04, 1.08)**
Rurality	Major cities	**1.06 (1.03, 1.09)**	**1.07 (1.04, 1.10)**	**1.04 (1.01, 1.07)**	**1.06 (1.04, 1.07)**
	Inner regional	1.02 (0.98, 1.07)	1.04 (0.99, 1.09)	1.04 (0.99, 1.09)	**1.03 (1.00, 1.06)**
	Outer regional	1.00 (0.94, 1.05)	1.06 (0.99, 1.13)	1.01 (0.94, 1.08)	1.02 (0.98, 1.05)
	Remote	1.00 (0.79, 1.25)	1.03 (0.80, 1.34)	1.16 (0.93, 1.46)	1.06 (0.91, 1.24)
	Very remote	0.87 (0.70, 1.07)	0.79 (0.61, 1.02)	1.01 (0.81, 1.25)	0.89 (0.77, 1.03)
IRSAD	High	1.02 (0.98, 1.07)	**1.04 (1.00, 1.10)**	**1.05 (1.00, 1.11)**	1.02 (0.98, 1.07)
	Middle	**1.05 (1.02, 1.08)**	**1.09 (1.05, 1.12)**	**1.05 (1.02, 1.09)**	**1.05 (1.02, 1.08)**
	Low	**1.05 (1.02, 1.08)**	**1.07 (1.03, 1.11)**	1.03 (0.99, 1.07)	**1.04 (1.02, 1.06)**

^*^Bold values indicate significant relative risk. Note: values are rounded to the second decimal place

Those who lived in major cities were at significantly higher risk of mortality during heatwaves than non-heatwaves (*RR* = 1.06, *CI*: 1.04–1.07), and this effect was greater in comparison to rural and regional areas across all severities. Those who lived in inner regional areas were at significantly higher risk of mortality during heatwaves in comparison to non-heatwave periods (*RR* = 1.03, *CI*: 1.00–1.06). Queenslanders from middle and low IRSAD deciles were at greater risk of mortality across all severities, apart from low IRSAD at extreme heatwaves which was not significant. However, Queenslanders from high IRSAD deciles were only at had greater risk of mortality during severe and extreme heatwaves (Table 1).

**Fig. 6:**
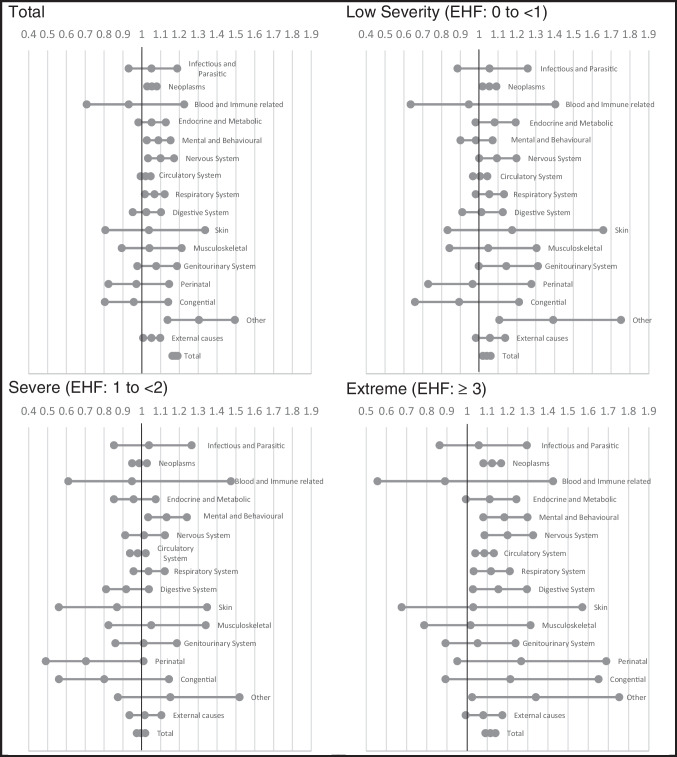
Relative risk by cause of death for matched days, overall, and by severity. Note: other includes the categories eye, ear, and mastoid; pregnancy and childbirth; and symptoms and other

The risk of mortality varied by the cause of death. The relative risk of dying from neoplasms (*RR* = 1.05, *CI*: 1.03–1.08), mental and behavioral conditions (*RR* = 1.09, *CI*: 1.03–1.15), nervous system (*RR* = 1.10, *CI*: 1.03, 1.17), respiratory system (*RR* = 1.07, *CI*: 1.02–1.12), and other underlying causes of death (*RR* = 1.30, *CI*: 1.14–1.49) increased during heatwave periods (Fig. 6). During low severity heatwaves, the risk of mortality was significantly greater for neoplasms (*RR* = 1.05, *CI*: 1.02, 1.09) and other (*RR* = 1.39, *CI*: 1.11–1.75). During severe heatwaves, the risk of mortality was significantly greater for mental and behavioral conditions (*RR* = 1.13, *CI*: 1.03–1.24). During extreme heatwaves, the risk of mortality was increased for neoplasms (*RR* = 1.12, *CI*: 1.08–1.17), mental and behavioral conditions (*RR* = 1.18, *CI*: 1.08–1.30), respiratory (*RR* = 1.12, *CI*: 1.03–1.21), and other (*RR* = 1.34, *CI*: 1.02–1.75) (Fig. 6).

## Discussion

The study identified that over the 10-year period from 2010 to 2019, heatwaves increased the risk of mortality in Queensland by 1.05 times. The size of this effect was varied across years, regions, ages, and heatwave severity. The numbers of heatwave days per annum in Queensland are increasing, from 9504 days in 2010/11 to 31,236 days in 2018/19 across 528 SA2 regions. As global climate change continues to increase the intensity and frequency of extreme heat events (Steffen et al. 2014), it is important to understand the impact of these events on the health of individuals across different environmental conditions and different social economic circumstances. This study explored the risk of mortality across various local circumstances using the state of Queensland, Australia as a case study.

### Heatwaves and age

There were population groups that were more impacted than others. Older adults above 60 years old were shown to be at an increased risk of mortality at varying severities of heatwaves. This finding is consistent with other Australian studies which have supported that the elderly are more vulnerable to heatwaves (Wang et al. 2012, 2015; Hansen et al. 2008; Pham et al. 2019; Schaffer et al. 2012; Tong et al. 2014b). On the other hand, the results highlighted a protective effect for children aged 0–4 years old during severe heatwaves. Other findings regarding children’s mortality during heatwaves are inconsistent, and a global literature review by Xu et al. (2012) suggested that heat-related deaths are higher in infants. In Adelaide, South Australia, Nitschke et al. (2011) found a significant mortality rise in 0–4 years old during the 2008 heatwaves. These inconsistencies highlight a need for further exploration of the impact of heatwave morality on 0–4 years old to identify sources of variability and to determine the best outcome measures to quantify the impact of heatwaves on children (Xu et al. 2014).

### Heatwaves and socioeconomic advantage

Queenslanders from middle and low IRSAD deciles were at greater risk of mortality across all severities, apart from low IRSAD at extreme heatwaves, which was not significant. However, Queenslanders from high IRSAD deciles were only at greater risk during extreme heatwaves. Though low socioeconomic status (SES) has been determined a risk factor for health service usage during heatwaves across Australia (Toloo et al. 2014; Xiao et al. 2017; Patel et al. 2019; Zhang et al. 2013), this effect has not been established for mortality in Queensland (Mason et al. 2022). The results warrant further investigations of SES as a risk factor for heatwave mortality in Queensland, and such investigations likely need to be combined with other measures such as type of housing, access to air conditioning, and tree cover.

### Heatwaves and rurality

Individuals living in major cities in Queensland were at a higher risk of mortality during heatwaves across all severities. In New South Wales, Jegasothy et al. (2017) found a similar effect, noting the rarity of extreme events and smaller populations in outer regional and remote areas which may influence statistical significance.

### Heatwaves and Aboriginal status

Aboriginal and Torres Strait Islander peoples were not found to be of higher risk of mortality during heatwave periods.

### Heatwaves and medical conditions

This study is consistent with others’ findings that those with respiratory, mental, and behavioral conditions were more likely to have been impacted by heatwaves (Mason et al. 2022). An unexpected finding was the significant relative risks for deaths due to neoplasm and nervous systems. This study used ICD10 codes to group to chapter levels and as such may miss some of the fidelity within the groupings where heatwaves have an impact, as all groups were impacted by heatwaves except for perinatal, blood and immune related, and congenital (World Health Organization 2015). Further work is also required to look at those who are more vulnerable, for example, those living with multiple health conditions as to date, we have only used the primary underlying cause of death to explore impact. Further investigations are required to explore sub-categories within demographic groups and causes of death to address high variability. Additionally, further research should explore non-death health outcomes during heatwaves to determine areas of greatest need.

Those at most risk are older populations, those from areas with greater disadvantage, and those in major cities. It should be noted that there was variability across the state, with some SA2 areas showing greater impact from heatwaves than others requiring further modeling to explore all the factors that may protect or increase the impact of heatwaves. It was also interesting to note the variability of the impact of heatwaves over time, indicating that there may be some learning that occurs within the communities and health systems to protect people or that there may be other factors coupled with heatwaves that cause excess mortality.

There may also be other factors at play that cause mortality during heatwaves, such as reduced air quality from bush fires. There may also be impacts form other environmental conditions including humidity. Further work is necessary to identify how these factors may vary the observable risk associated with heatwaves.

### Policy implications

Heatwaves significantly impact the risk of death in Queensland; however, heatwaves are not explicitly mentioned in the Disaster Management Act 2003 (Queensland Government 2003), and the state lacks a heatwave early warning system. Policy should reinforce the significant risk that comes with prolonged periods of heat, and such events should be quantified in terms of other disasters. Vulnerable populations within communities must be protected if we want heatwave deaths to fall in the wake of increased heat due to climate change.

The Intergovernmental Panel on Climate Change has identified the need for political commitment and follow through to accelerate climate change adaptation actions (Intergovernmental Panel on Climate Change 2022). The Queensland Government has identified that climate change is a risk (Chesnais et al. 2019). As a responsibility of government is to work to protect its people, this should include protecting people from the impacts of increased heat (Harper 2020). With variation in the impacts of heat on deaths across Queensland, policy at a statewide level must enable local action. State-wide policy should involve the consideration of the most vulnerable, community needs, and the timing of wider work, recreation, and sporting activities. Adaptation and sustainable mitigation strategies to reduce the lives lost due to heatwaves should be explored in the future research.

### Strengths and limitations

The key strength of this study is that it used population-based data to explore heatwaves and mortality across the whole of Queensland, for all deaths, over a 10-year period. However, a retrospective case-matching approach was undertaken, which does not allow the establishment of causal relationships. Flagging heatwave events, along with other disasters within the deaths data, would improve future explorations of heatwave deaths in Queensland.

Cases (deaths) were matched using their usual area of residence. It is possible that an individual may have died outside of their usual area of residence and meteorological data were not adequately matched to the region they died in. Furthermore, prior to matching, heatwave days and date of death were converted to day of the year from 1 to 366, which were offset by 1 day in 2012 and 2016 due to leap years. This study determined crude relative risk only, and confounding variables were not adjusted for in the analysis. Future studies should adjust for factors such as humidity and air quality, to improve the internal validity of the results.

Heatwaves may impact mortality in the days following a heatwave event, and these effects may not have been captured in the data. For example, heatwaves may trigger cardiovascular symptoms that lead to death days later (lag days). Heatwaves may also result in a cascade of extreme weather events including drought and flooding (Sutanto et al. 2020; Nairn et al. 2021), resulting in further increased morbidity and mortality, which was not explored in this study. Additionally, this study explored risk based on cause of death coded by the ICD-10. Future investigations should explore underlying causes of death and multimorbidity.

## Conclusion

This study found that overall, Queenslanders are at greater risk of mortality during heatwave periods. Those who are at greatest risk, including people living in major cities, in low socioeconomic regions, the elderly, and those with medical conditions, must be protected to reduce lives lost to heatwaves. Further investigations must be undertaken to establish a causal link between heatwaves and deaths in Queensland, and to determine patterns of non-death outcomes across the state. Without appropriate government action and policy, it is expected that heatwave deaths will continue to increase in the wake of increased extreme heat due to climate change.

## Supplementary Information

Below is the link to the electronic supplementary material.

Supplementary file1 (DOCX 16142 KB)

## Data Availability

Data is available via the Australian Bureau of Statistics once ethical approval for the use of the data has been granted.
