# Supplementary material for: Heatwaves and mortality in Queensland 2010–2019: implications for a homogenous state-wide approach
Source: Int J Biometeorol. 2023 Feb 3;67(3):503–15. doi: 10.1007/s00484-023-02430-6 (PMC9974727; doi:10.1007/s00484-023-02430-6)

**Supplementary File 1- Heatwave maps 2010/11 to 2018/19**

1. Heatwave map 2010/11


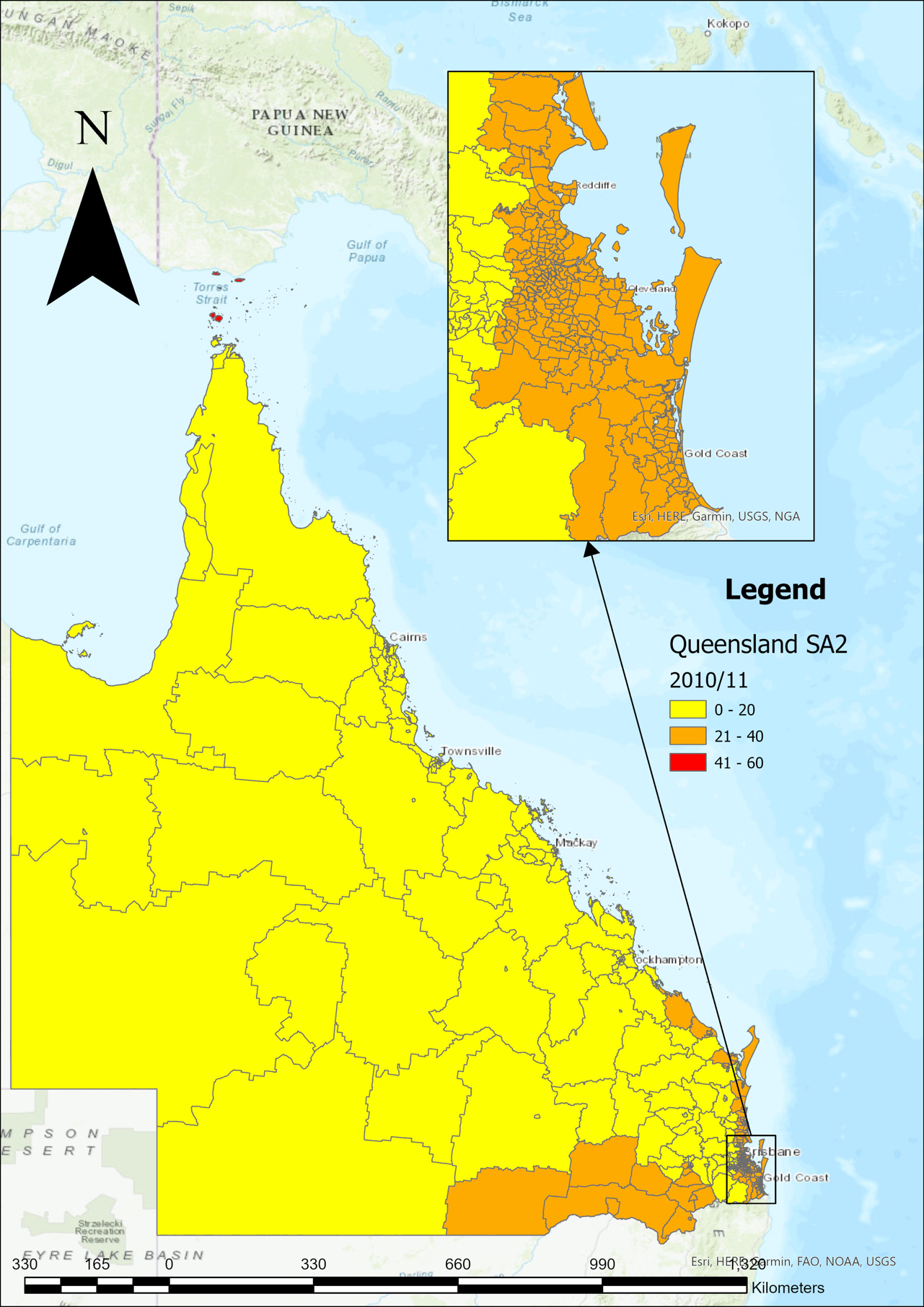


1. Heatwave map 2011/12


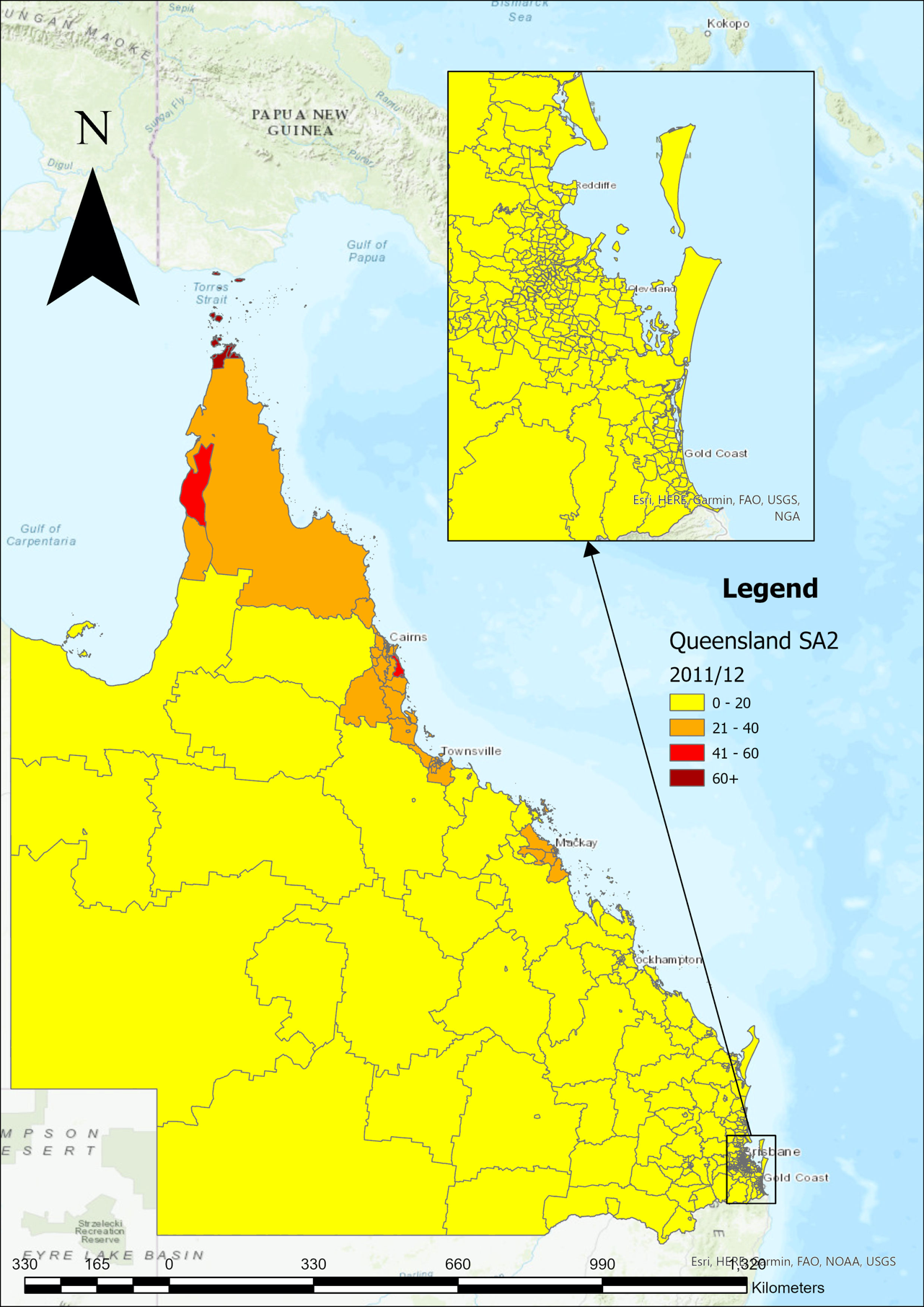


1.
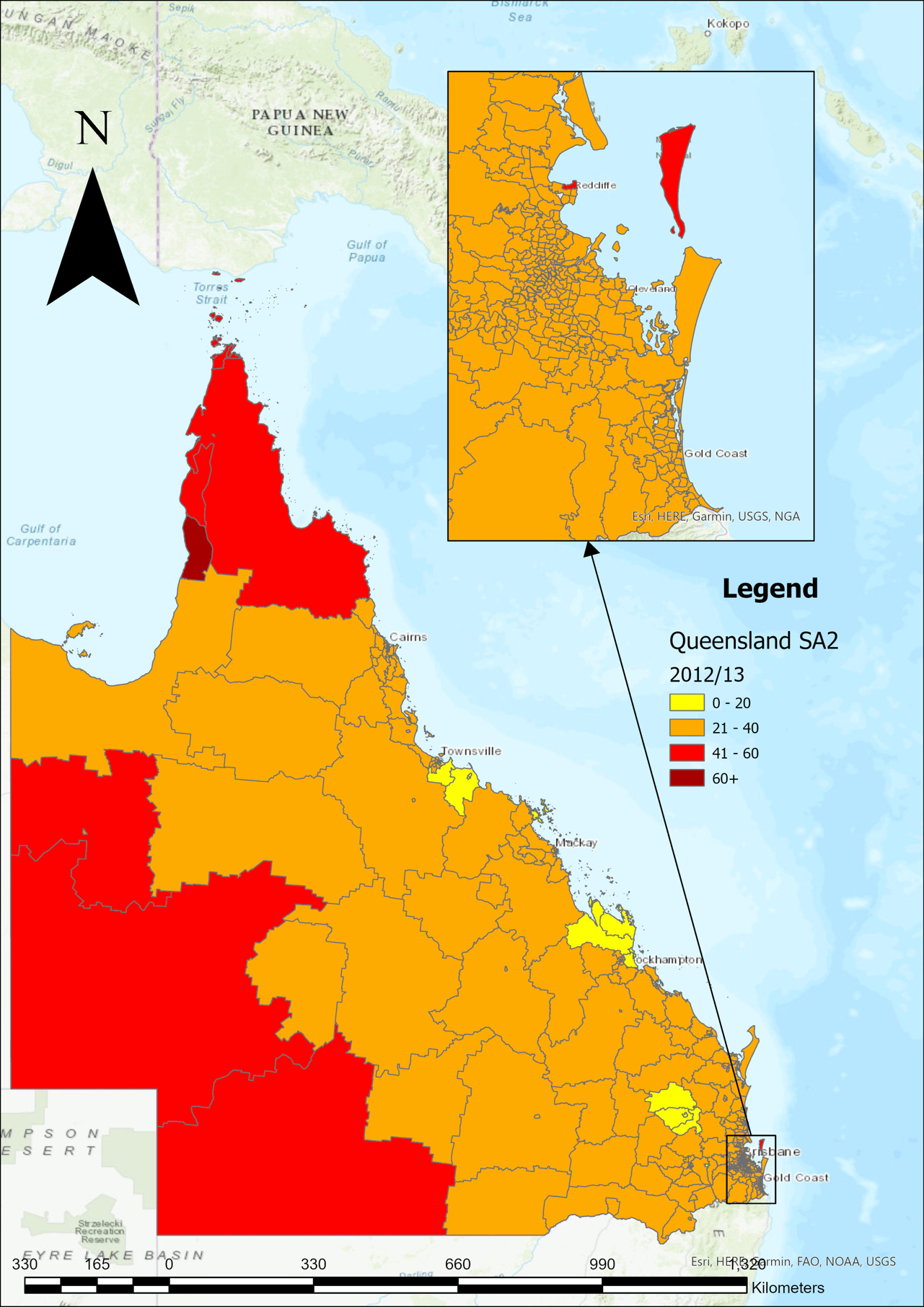
Heatwave map 2012/13
2. Heatwave map 2013/14


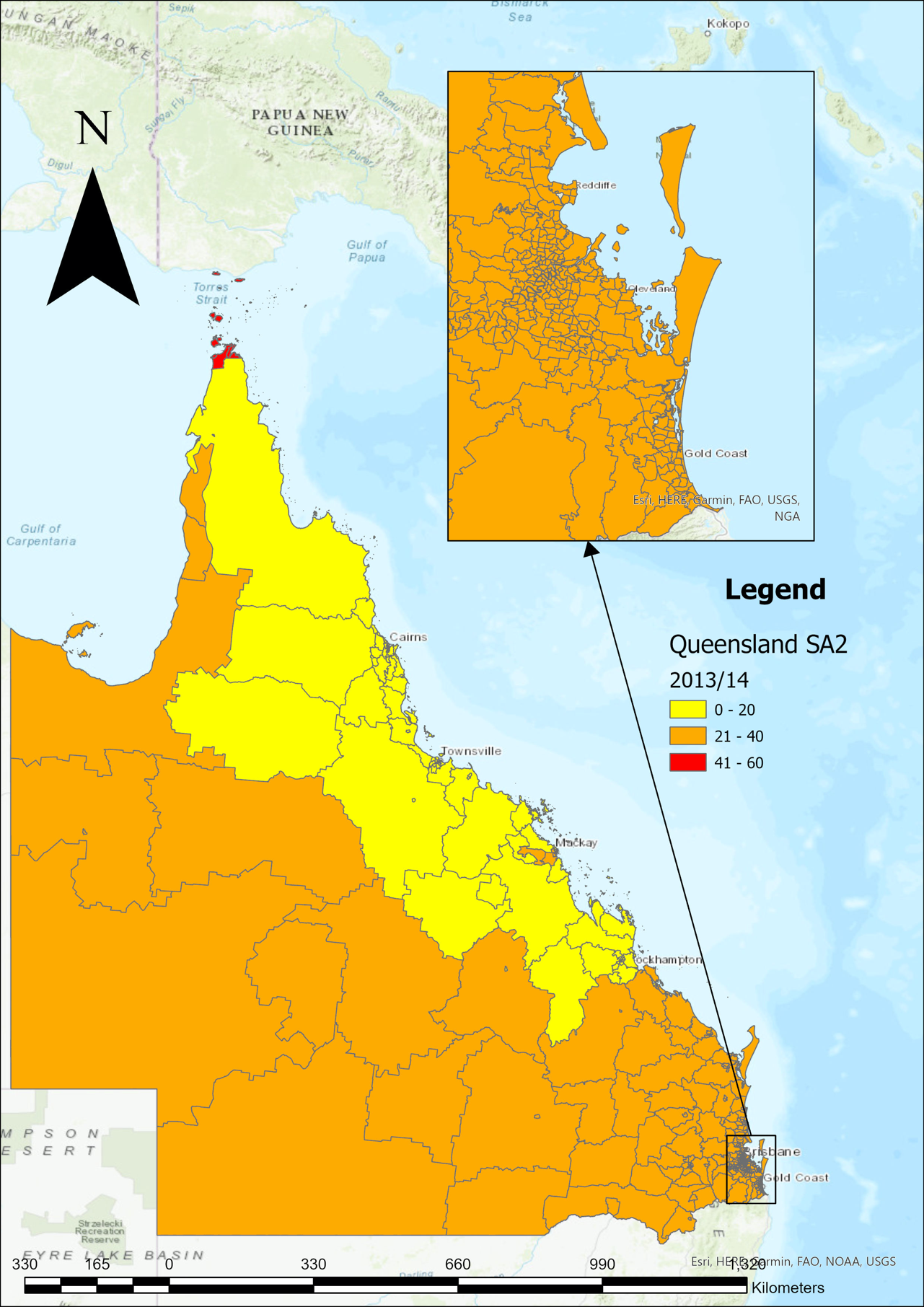


1.
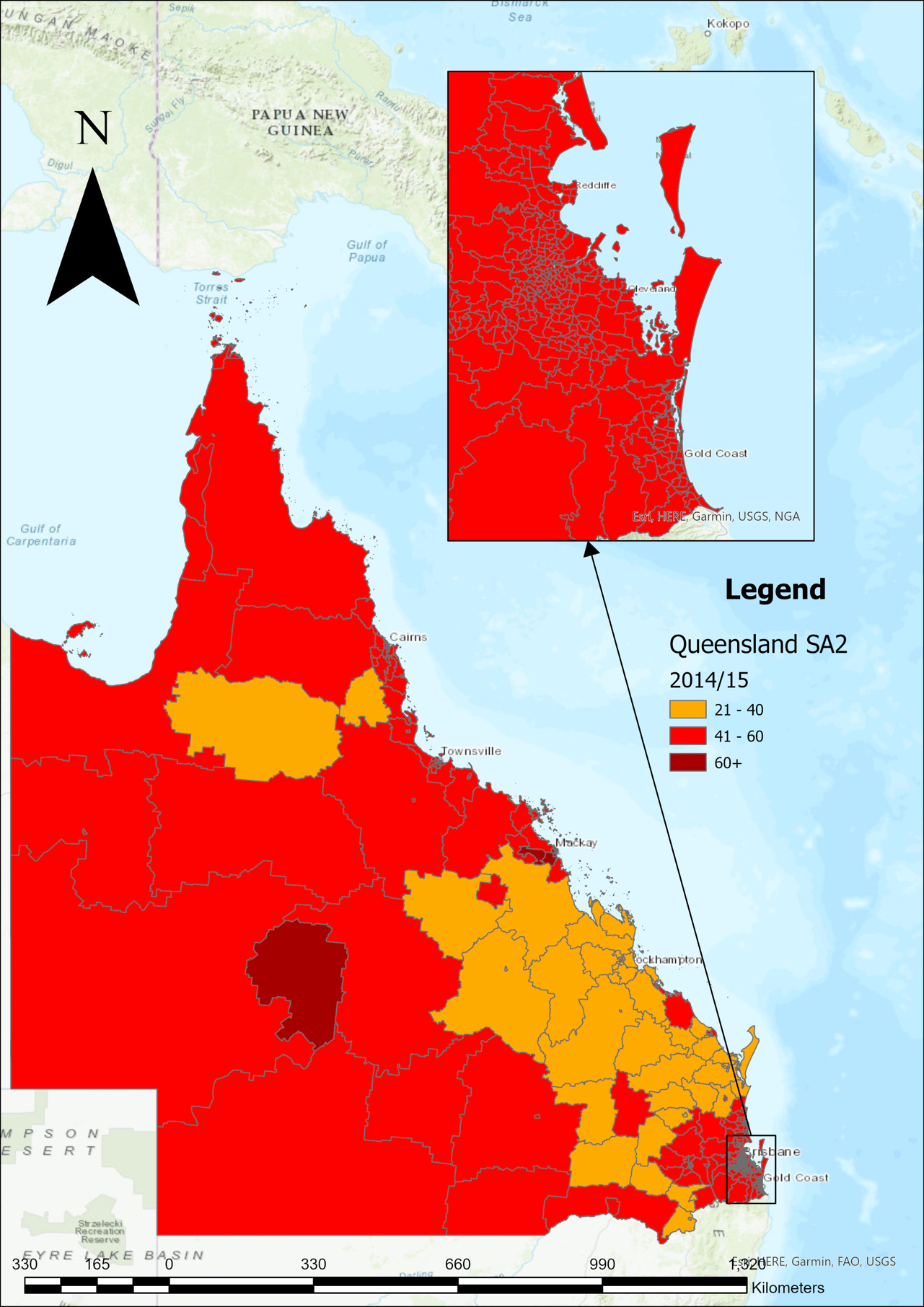
Heatwave map 2014/15
2. Heatwave map 2015/16


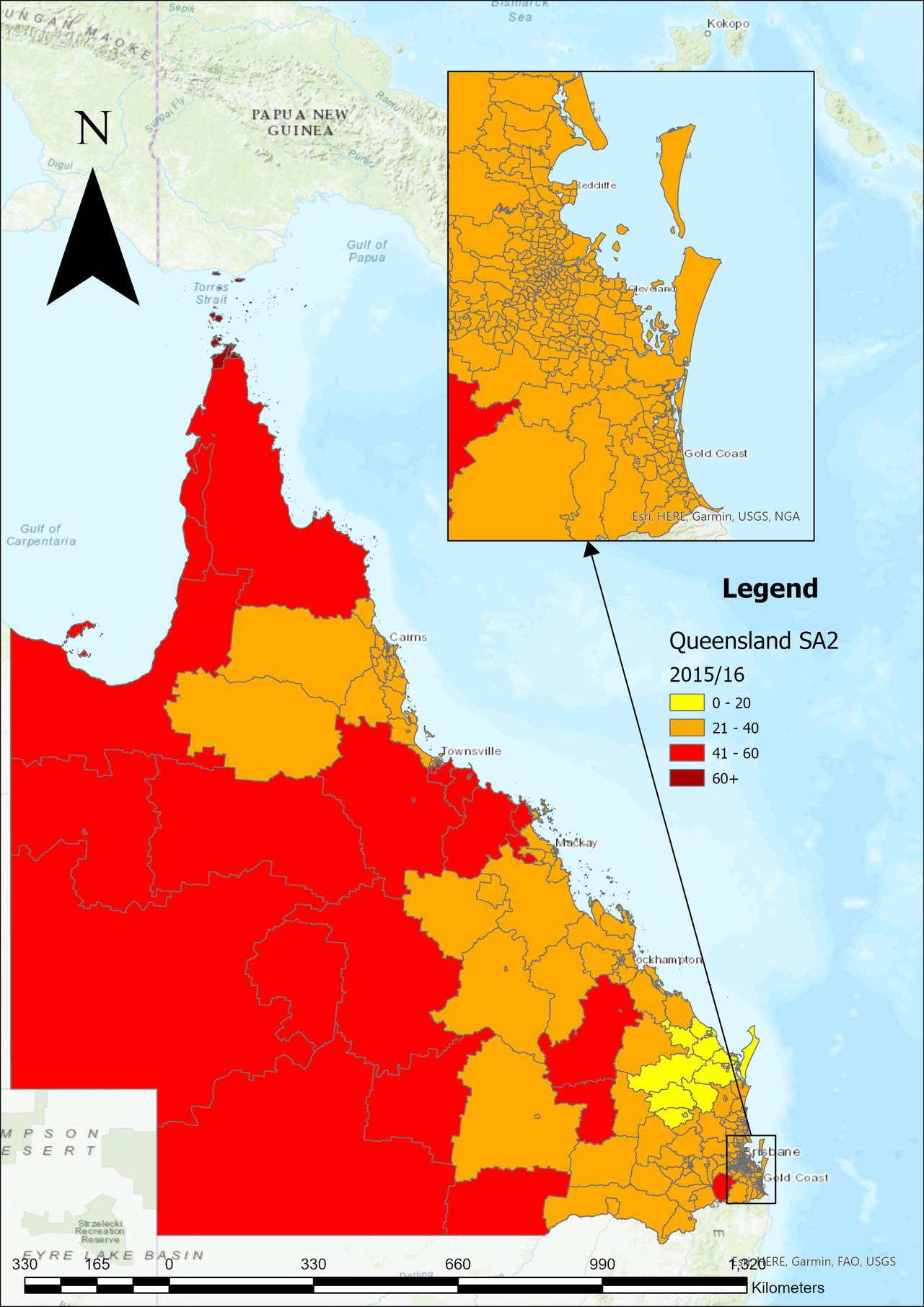


1. Heatwave map 2016/17


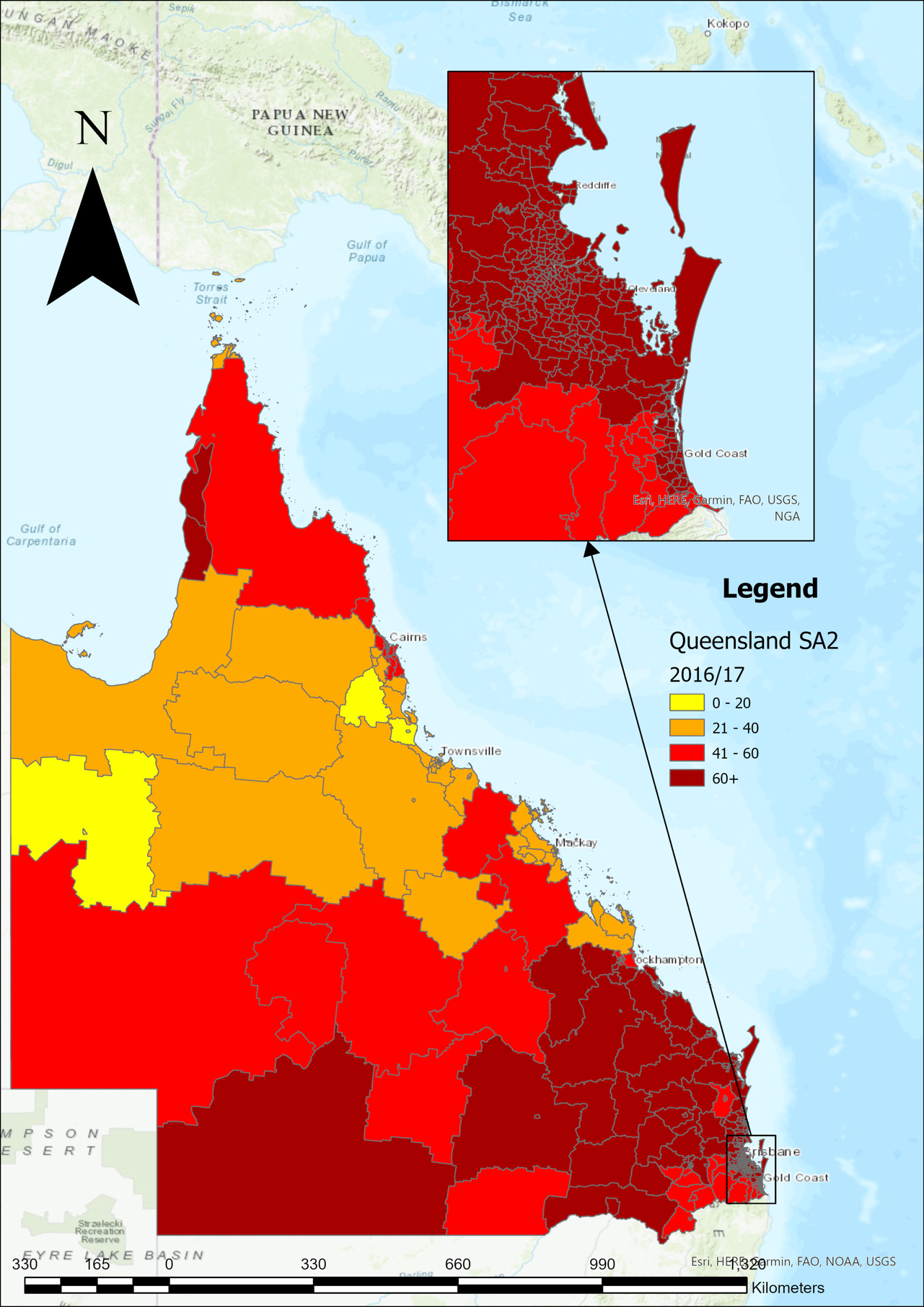


1.
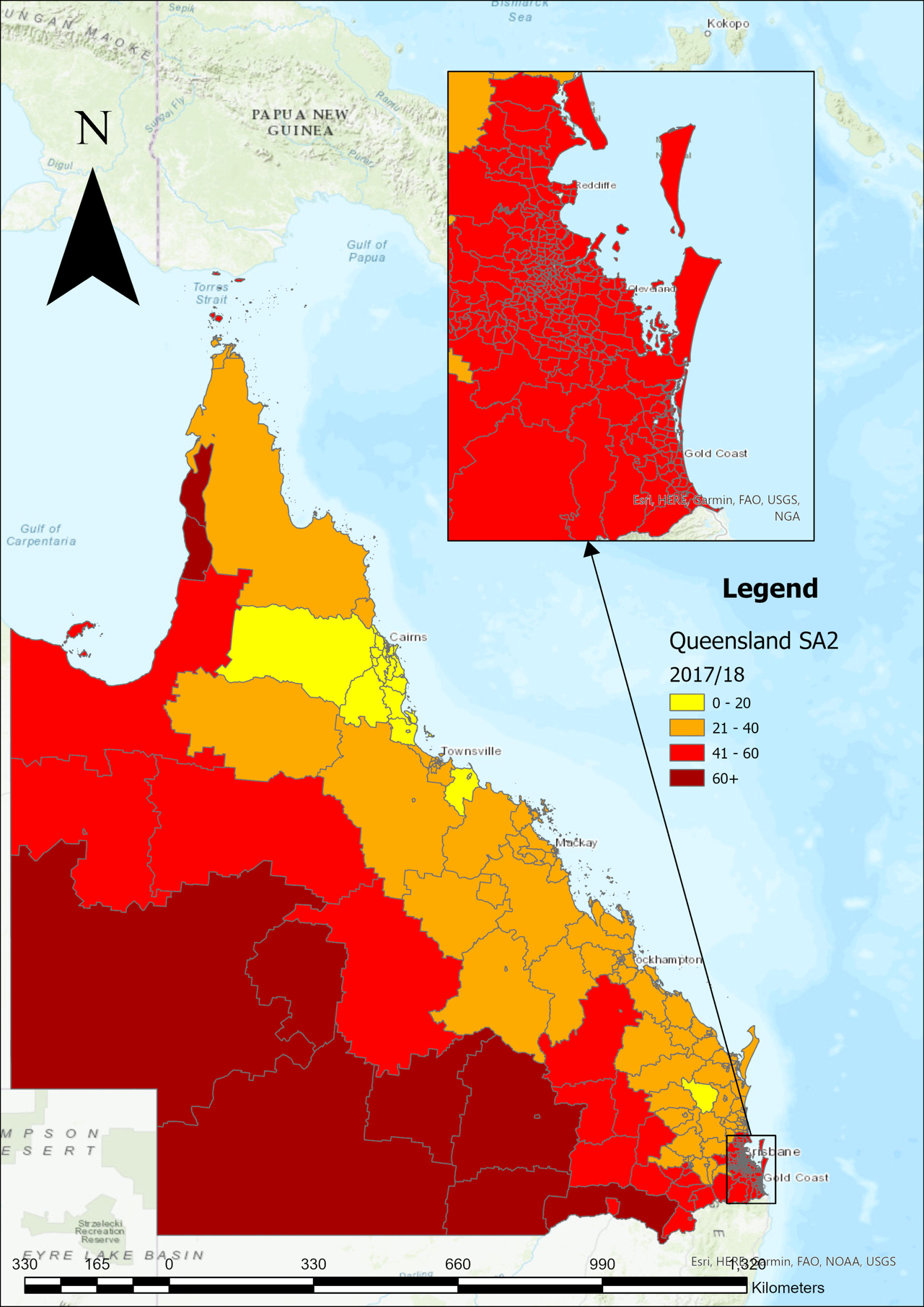
Heatwave map 2017/18
2. Heatwave map 2018/19


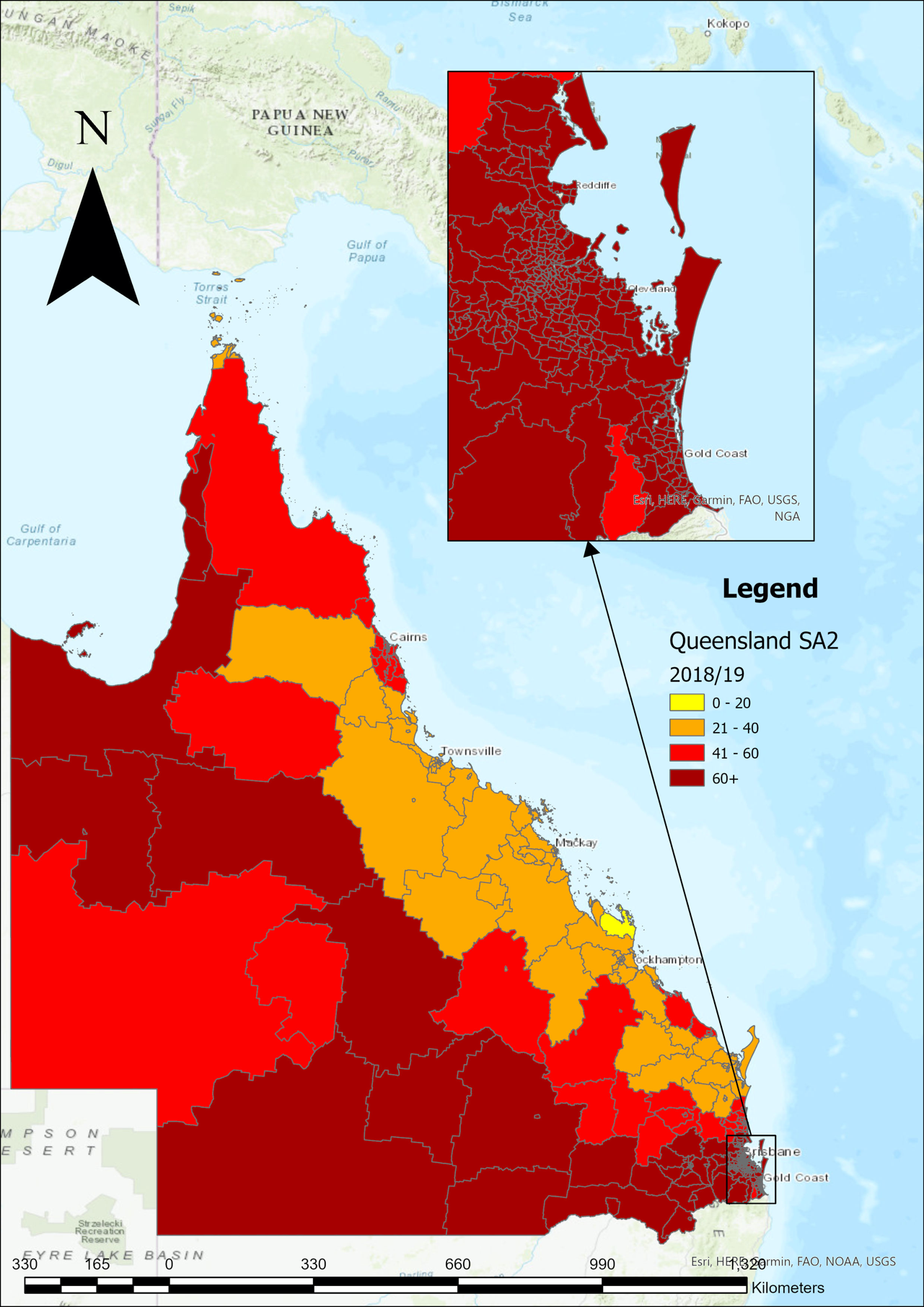

Supplement: Supplementary file 1 — Supplementary file1 (DOCX 16142 KB) [file 484_2023_2430_MOESM1_ESM.docx]
